# Genome-Wide Identification and Tissue-Specific Expression Analysis of the *FtAQP* Gene Family in Tartary Buckwheat (*Fagopyrum tataricum*)

**DOI:** 10.3390/genes17040479

**Published:** 2026-04-17

**Authors:** Wenxuan Chu, Zhikun Li, Ziyi Zhang, Yutong Zhu, Yan Zeng, Ruigang Wu, Xing Wang

**Affiliations:** School of Landscape and Ecological Engineering, Hebei University of Engineering, Handan 056038, China

**Keywords:** aquaporins (AQPs), genome-wide identification, abscisic acid, expression pattern analysis

## Abstract

Background: Tartary buckwheat (*Fagopyrum tataricum*) serves as an excellent model for studying plant water adaptation mechanisms due to its exceptional drought tolerance. While aquaporins (AQPs) mediate the transmembrane transport of water and solutes in plants, their fine-tuned regulatory networks underlying stress resilience in Tartary buckwheat remain largely elusive. Methods: Here, we combined bioinformatics and transcriptomics to systematically identify 30 highly conserved *FtAQP* genes at the genome-wide level. Results: Cross-validated by qRT-PCR, our analysis revealed their distinct expression patterns across different organs. Based on our transcriptomic data, we hypothesize that *FtAQP* family members potentially participate in a coordinated whole-plant water management network through differential spatiotemporal expression. Specifically, the robust transcription of *FtAQP8*, *FtAQP12*, and *FtAQP28* in roots is associated with the initial water uptake process. As water undergoes long-distance transport, the synergistic upregulation of *FtAQP13*, *FtAQP17*, *FtAQP20*, and *FtAQP29* in the stem suggests a potential role in facilitating critical lateral water flow. Furthermore, during reproductive development, *FtAQP27* exhibits extreme tissue specificity in floral organs, implying its possible involvement in maintaining local osmotic homeostasis. Furthermore, the promoter regions of *FtAQPs* are highly enriched with cis-acting elements responsive to light, abscisic acid (ABA), and cold stress, suggesting they are intimately regulated by a coupling of endogenous phytohormones and environmental cues. Conclusions: Ultimately, this study provides valuable insights into the potential molecular basis of multidimensional water regulation in Tartary buckwheat, and identifies candidate genetic targets for improving water use efficiency in dryland agriculture through the precise manipulation of aquaporins. Collectively, while these observational findings provide valuable predictive models, future in vivo experimental validations are required to confirm their exact biological functions.

## 1. Introduction

Plant aquaporins (AQPs) are channel proteins localized on the cell membrane, constituting critical pathways for the highly efficient transmembrane transport of water and certain small-molecule solutes [1,2,3]. As a large and functionally diverse protein family, they regulate the entire water flow pathway within plants—from root water uptake and long-distance transport to leaf transpiration—and also participate in the transport of various small-molecule solutes, including carbon dioxide, ammonia, urea, boron, and silicon [4]. Given that AQPs play a certain role in regulating plant water relations, AQPs serve as critical nodes in the signaling and physiological networks mediating plant responses to external water stress [5,6]. Structurally, plant AQPs exhibit highly conserved features. A single AQP polypeptide chain typically comprises six transmembrane helices and a characteristic “NPA” (asparagine-proline-alanine) motif; the water-selective pore formed by this motif is the core of its function [7,8,9]. Based on sequence homology, plant AQPs are divided into several subfamilies. The prevalent Plasma Membrane Intrinsic Proteins (PIPs) serve as the primary sites for water exchange between cells and the external environment, directly participating in critical processes such as root water uptake and the regulation of stomatal movement [2,6]. Tonoplast Intrinsic Proteins (TIPs) are primarily localized to the vacuolar membrane and are responsible for regulating water exchange between the cytoplasm and the vacuole, which is essential for maintaining cell turgor and osmotic regulation [7,10]. Members of the Nodulin-26-like Intrinsic Protein (NIP) subfamily display the most diverse substrate selectivity. In addition to water, they can transport glycerol, urea, and ammonia, as well as metalloids like boron, silicon, and arsenic, playing unique roles in plant nutrient uptake and detoxification [2,4]. Beyond regulating water balance, the physiological significance of AQPs extends to facilitating gas exchange and photosynthesis through the transport of various small molecules. Certain PIP subfamily members have been proven to conduct carbon dioxide. By promoting the influx of CO_2_ from the intercellular spaces into the chloroplasts of mesophyll cells, these AQPs potentially enhance photosynthetic efficiency, thereby indirectly influencing plant growth and biomass accumulation [5,11,12,13].

In recent years, driven by the increasing availability of high-quality genome assemblies, the genome-wide identification and functional characterization of the AQP family have been extensively conducted in model plants and various major food crops. For instance, the AQP families in species such as sorghum (*Sorghum bicolor*), bread wheat (*Triticum aestivum*), and oat (*Avena sativa*) have been systematically identified. Studies demonstrate that specific AQP members in these crops significantly enhance plant stress resilience by responding to drought stress signals [13,14,15]. However, current research on the AQP gene family predominantly focuses on typical gramineous crops or model plants. For non-model pseudocereals that have evolved extreme drought tolerance mechanisms over long-term evolution, the molecular mechanisms by which their AQP families coordinate whole-plant water homeostasis through spatial distribution and the divergence of stress-responsive elements remain largely elusive.

Tartary buckwheat (*F. tataricum*) inherently possesses robust drought resistance, and its drought adaptation mechanisms involve a multitude of physiological and biochemical regulations as well as gene expression alterations. Although AQPs generally play a pivotal role in plant drought resistance—such as regulating root water uptake, stomatal movement, and cellular water potential—investigating how specific members of the AQP family directly participate in these stress responses in Tartary buckwheat is of paramount significance [16,17,18,19,20].

## 2. Materials and Methods

### 2.1. Identification of the FtAQP Gene Family in Tartary Buckwheat

The whole-genome sequence and corresponding gene annotation files of Tartary buckwheat (*F. tataricum*) were retrieved from the MBKbase database (http://www.mbkbase.org/Pinku1/, accessed on 25 March 2025). The AQP protein sequences of the model plant *Arabidopsis thaliana* were obtained from the TAIR database (https://www.arabidopsis.org/, accessed on 25 March 2025) to serve as queries. A BLASTP search was performed against the Tartary buckwheat protein database using TBtools (Version 2.388), with an E-value threshold set to 1 × 10^−5^. To confirm the accuracy of the identification, all candidate sequences were verified for the presence of the conserved Major Intrinsic Protein (MIP) domain. InterPro scan results (https://www.ebi.ac.uk/interpro/entry/pfam/PF00230/, accessed on 25 March 2025) confirmed that all screened members possess the characteristic Pfam domain PF00230, which belongs to the Aquaporin-like clan. Consequently, these validated sequences were definitively identified as *FtAQP* gene family members [21,22].

### 2.2. Analysis of Physicochemical Properties and Secondary Structure Prediction

The physicochemical properties of FtAQP proteins were analyzed using the ExPASy ProtParam tool (https://web.expasy.org/protparam/, accessed on 25 March 2025). Key parameters evaluated included the number of amino acids, molecular weight (MW), theoretical isoelectric point (pI), total number of negatively and positively charged residues, instability index, aliphatic index, and grand average of hydropathicity (GRAVY) [2].

Secondary structure prediction was performed using the SOPMA tool available on the Prabi server (https://npsa-prabi.ibcp.fr/cgi-bin/npsa_automat.pl?page=/NPSA/npsa_sopma_f.html, accessed on 25 March 2025). The structural components analyzed included α-helix, extended strand, β-turn, and random coil [23].

### 2.3. Chromosomal Distribution and Phylogenetic Tree Construction

The chromosomal locations of the identified *FtAQP* genes were mapped using the “Gene Location Visualize from GTF/GFF” function within the TBtools software.

For phylogenetic analysis, multiple sequence alignment of the FtAQP protein sequences was performed using ClustalW integrated into MEGA 11.0. Subsequently, a phylogenetic tree was constructed using the Maximum Likelihood (ML) method with 1000 bootstrap replicates to ensure node reliability. The resulting phylogenetic tree was visualized and annotated using the iTOL online server (https://itol.embl.de/, accessed on 25 March 2025) [24,25].

### 2.4. Analysis of Conserved Motifs and Gene Structure

The conserved motifs of the FtAQP proteins were analyzed using the MEME online suite (Version 5.5.7; https://meme-suite.org/meme/tools/meme, accessed on 25 March 2025). The maximum number of motifs to be identified was set to 10, while all other parameters were maintained at default settings [12]. Subsequently, the gene structure (exon-intron organization) and conserved motif composition of the candidate *FtAQP* members were visualized using the TBtools software [26].

### 2.5. Prediction of Cis-Acting Elements

To analyze the promoter regions of the *FtAQP* genes, the genomic sequences 2000 bp upstream of the transcription start site (TSS) were extracted using TBtools. These upstream sequences were then submitted to the PlantCARE database (https://bioinformatics.psb.ugent.be/webtools/plantcare/html/, accessed on 25 March 2025) to identify putative cis-acting regulatory elements. The predicted elements were classified and visualized using TBtools [27].

### 2.6. Synteny and Collinearity Analysis

The synteny relationships of *FtAQP* genes within the Tartary buckwheat genome were analyzed and visualized using the Advanced Circos module in TBtools.

### 2.7. Analysis of Tissue-Specific Expression Patterns

Publicly available gene expression data for different tissues of Tartary buckwheat were retrieved from the NCBI Gene Expression Omnibus (GEO) database (https://www.ncbi.nlm.nih.gov/geo/query/acc.cgi?acc=GSE126576, accessed on 25 March 2025). These data were used to analyze the tissue-specific expression profiles of the *FtAQP* gene family. The expression patterns were subsequently visualized by constructing heatmaps using the TBtools software.

### 2.8. Plant Materials

The experiment was conducted in May 2025 at Hebei University of Engineering using the Tartary buckwheat cultivar ‘Xiqiao No. 2’. At the flowering stage, 30 plants exhibiting uniform growth were selected and divided into three groups to serve as biological replicates. Samples were collected from four tissues: roots, stems, leaves, and flowers. All samples were immediately frozen in liquid nitrogen and stored at −80 °C for subsequent transcriptome analysis.

### 2.9. Quantitative Expression Analysis by RT-qPCR

To validate the reliability of our RNA-seq data, the relative expression levels of 12 selected differentially expressed *FtAQP* genes were analyzed using quantitative real-time PCR (qRT-PCR). Gene-specific primers for qRT-PCR were designed using Primer-BLAST (https://www.ncbi.nlm.nih.gov/tools/primer-blast, accessed on 25 May 2025) and are listed in Table 1. The *FtActin* gene was selected as the internal control, as its expression stability in Tartary buckwheat has been validated in previous studies [28]. Furthermore, prior to formal quantification, the amplification efficiency of all primer pairs (including the reference gene) was rigorously evaluated using a standard curve generated from a 5-fold serial dilution of cDNA templates. The results confirmed that the amplification efficiencies ranged from 95% to 105%, with correlation coefficients (R^2^) > 0.99, demonstrating their suitability for the 2^−ΔΔCT^ method. Using total RNA extracted from various Tartary buckwheat tissues as templates, first-strand cDNA was synthesized using the PrimeScript cDNA Synthesis Kit (Takara, Dalian, China) according to the manufacturer’s instructions. Subsequently, a 20 μL reaction mixture was prepared, comprising 1 μL of cDNA template, 1.5 μL of primer mix (15 pmol/μL), 10 μL of qPCR Master Mix (Takara, Dalian, China), and 7.5 μL of sterile water. The qRT-PCR assays were performed on a CFX Connect Real-Time PCR Detection System (Bio-Rad, Hercules, CA, USA) with the following thermocycling program: initial denaturation at 95 °C for 30 s, followed by 40 cycles of 95 °C for 5 s and 60 °C for 34 s, and a final step at 72 °C for 10 s. After amplification, a melting curve analysis from 65 °C to 95 °C was performed to verify the specificity of the PCR products. All reactions were conducted with three biological and three technical replicates. The relative expression level of each gene was calculated using the 2^−ΔΔCT^ method, normalized to the internal reference gene. Finally, the Pearson correlation coefficient between the RNA-seq and qRT-PCR data was calculated, and a linear regression scatter plot was generated to evaluate their consistency [29].

Statistical analysis and data visualization were performed using Python (version 3.12) leveraging the scientific computing ecosystem including Pandas, NumPy, Matplotlib, Seaborn, and Scikit-learn.

## 3. Results

### 3.1. Physicochemical Properties and Secondary Structure Prediction of the FtAQP Gene Family Members

A total of 30 AQP gene family members were identified in the Tartary buckwheat genome and designated as *FtAQP1* through *FtAQP30* (Table 2). The length of the encoded proteins ranged from 198 to 311 amino acids (aa). The molecular weights (MWs) varied from 20,842.33 to 32,415.56 Da, with an average of 28,260.56 Da. Analysis of the isoelectric point (pI) showed that 20 members were basic (pI > 7), while 10 were acidic (pI < 7). The total numbers of negatively and positively charged residues ranged from 5 to 19 and 7 to 21, respectively. The instability index ranged from 21.36 to 40.92. Notably, 29 members exhibited an instability index lower than 40, indicating that the majority of FtAQP proteins are stable. The aliphatic index ranged from 96.25 to 119.08, and the grand average of hydropathicity (GRAVY) varied from 0.365 to 0.875. Secondary structure prediction revealed that the FtAQP proteins were primarily composed of α-helices (23.16–50.62%), followed by random coils (21.40–42.12%), extended strands (18.36–35.46%), and β-turns (6.89–12.85%) (Table 3).

### 3.2. Chromosomal Distribution of the FtAQP Gene Family Members

**Figure 1:**
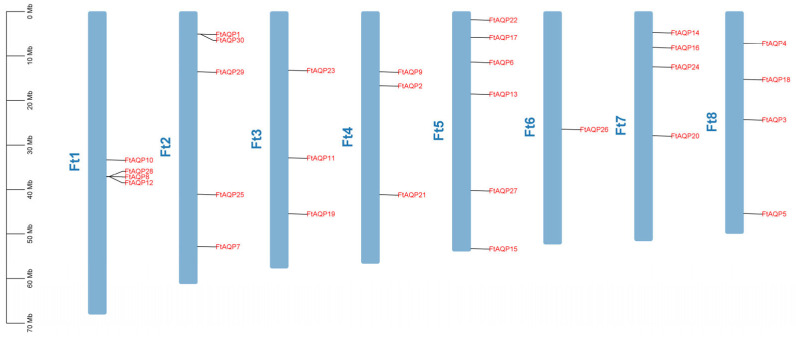
Chromosomal localization of the *FtAQP* gene family members. The physical location map was drawn based on the location of the *FtAQP* genes on the chromosomes by TBtools.

Chromosomal mapping revealed that the *FtAQP* gene family members were unevenly distributed across chromosomes 1 through 8. Chromosome 5 contained the largest number of genes, harboring six members. In contrast, Chromosome 6 had the fewest, containing only a single member (*FtAQP26*). Chromosome 2 had five members (*FtAQP1*, *FtAQP7*, *FtAQP25*, *FtAQP29*, and *FtAQP30*). Chromosomes 3 and 4 each contained four members, and Chromosomes 1, 7, and 8 similarly harbored three members each (Figure 1). These results demonstrated that while the *FtAQP* gene family members were distributed across all eight chromosomes, their distribution density is uneven.

### 3.3. Phylogenetic Relationship Analysis of the FtAQP Gene Family Members

**Figure 2:**
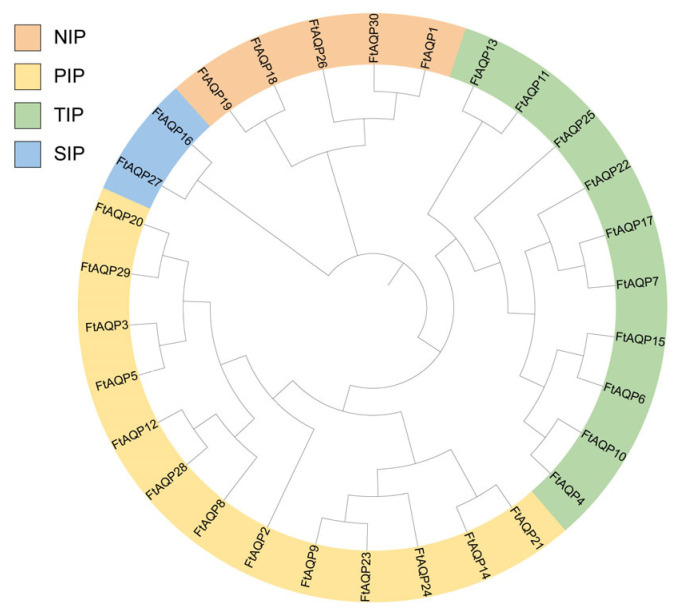
Phylogenetic analysis of the FtAQP proteins. The phylogenetic tree indicates that the FtAQP proteins are clustered into four distinct subfamilies: NIP, PIP, TIP, and SIP. The subfamilies are color-coded as follows: NIP (orange), PIP (yellow), TIP (green), and SIP (blue).

The phylogenetic relationships of the FtAQP proteins are illustrated in Figure 2. The *FtAQP* gene family was classified into four distinct subfamilies: NIP, PIP, TIP, and SIP. Specifically, the PIP subfamily contained the largest number of members (13), followed by the TIP subfamily with 10 members. The NIP subfamily contained 5 members, while the SIP subfamily was the smallest, containing only 2 members.

Furthermore, 12 paralogous gene pairs were identified within the *FtAQP* family: *FtAQP1*/*FtAQP30*,*FtAQP3*/*FtAQP5*,*FtAQP4*/*FtAQP10*,*FtAQP11*/*FtAQP13*, *FtAQP14*/*FtAQP21*,*FtAQP16*/*FtAQP27*,*FtAQP18*/*FtAQP19*,*FtAQP7*/*FtAQP17*, *FtAQP6*/*FtAQP15*, *FtAQP9*/*FtAQP23*, *FtAQP12*/*FtAQP28*, and *FtAQP20*/*FtAQP29*. Paralogous gene pairs are generally hypothesized to function coordinately in complex biological processes, such as signal transduction, metabolism, and developmental regulation, thereby enhancing the efficiency, precision, and robustness of these pathways.

### 3.4. Analysis of Conserved Motifs and Gene Structure of the FtAQP Gene Family Members

**Figure 3:**
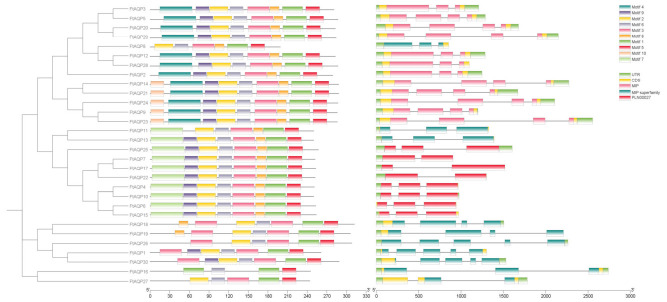
Gene structure and conserved motif analysis of the *FtAQP* gene family. The MEME URL was used for the motifs analysis of the 30 FtAQP protein sequences. Intron/exon structure of *FtAQP* genes was analyzed by TBtools. Gene models are drawn to scale, as shown in the bar at the bottom.

Based on the Tartary buckwheat proteome database, the conserved motifs of the FtAQP proteins were analyzed. As shown in Figure 3, the vast majority of *FtAQP* members contained Motifs 1, 2, 3, 4, 5, and 6. Among them, Motifs 1, 2, and 3 were distributed across almost all members, constituting the core structural framework of the AQP family. Members such as *FtAQP3*, *FtAQP5*, *FtAQP20*, *FtAQP29*, *FtAQP8*, *FtAQP12*, and *FtAQP28* generally harbored Motifs 1 and 5 at their N-terminus or within the central region of the sequence. In contrast, members including *FtAQP11*, *FtAQP13*, *FtAQP25*, *FtAQP7*, *FtAQP17*, and *FtAQP22* exhibited a relatively simplified motif pattern, primarily consisting of Motifs 7, 9, 2, 6, 3, 1, and 5. Furthermore, *FtAQP18*, *FtAQP19*, and *FtAQP26* displayed unique motif arrangements, featuring the repetition or specific displacement of Motif 3.

Domain analysis revealed that all *FtAQP* sequences possessed the typical Major Intrinsic Protein (MIP) superfamily domain (MIP superfamily/PLN00027). The sequence span of this domain was highly consistent with the distribution of the primary conserved motifs, strongly supporting their classification as aquaporins.

Regarding gene structure, the number of introns in the *FtAQP* genes ranged from 0 to 4. Genes such as *FtAQP11*, *FtAQP13*, *FtAQP25*, *FtAQP7*, *FtAQP17*, and *FtAQP22* were characterized by fewer and shorter introns. The total genomic span varied significantly among the members, ranging from less than 1000 bp to over 3000 bp. Notably, *FtAQP16* possessed the longest intron structure, resulting in a genomic length of nearly 3000 bp. While complete 5′ and 3′ untranslated regions (UTRs) were identified flanking the coding sequences (CDS) in most genes, members such as *FtAQP6*, *FtAQP15*, and *FtAQP1* lacked clearly defined UTR regions under the current genome annotation.

### 3.5. Prediction of Cis-Acting Elements in the Promoters of FtAQP Gene Family Members

**Figure 4:**
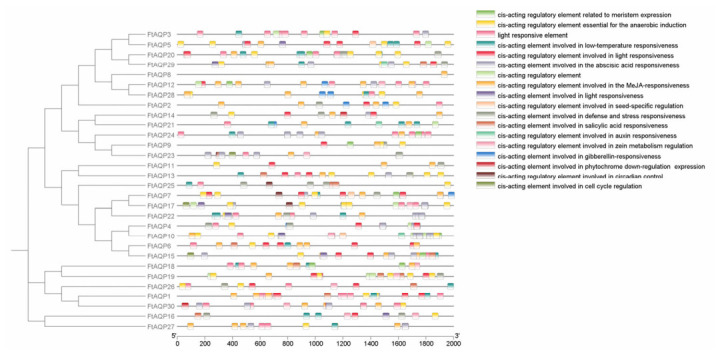
Analysis of *cis*-acting regulatory elements in the *FtAQP* gene family. The distribution of diverse predicted *cis*-acting elements within the 2000 bp upstream regions is shown alongside the phylogenetic tree of the FtAQP proteins. The specific functional categories of these elements are detailed in the right-hand key.

A systematic analysis of the cis-acting regulatory elements within the 2000 bp promoter region upstream of the transcription start site (TSS) of the *FtAQP* family members was performed using the PlantCARE online database (Figure 4). The results revealed that the promoter regions of this gene family are highly enriched with various regulatory elements, which can be broadly classified into four major categories: light responsiveness, hormone responsiveness, abiotic stress responsiveness, and plant growth and development regulation.

Light-responsive elements were the most widely distributed and abundant type within the *FtAQP* promoters. All analyzed *FtAQP* genes contained at least one light-responsive element in their promoter regions. Notably, members such as *FtAQP1*, *FtAQP6*, and *FtAQP23* exhibited a dense distribution of these elements.

Furthermore, various elements related to plant hormone signal transduction were identified, suggesting that *FtAQP* expression may be regulated by multiple hormones. Abscisic acid (ABA) responsive elements were widely distributed in members such as *FtAQP10*, *FtAQP30*, *FtAQP4*, and *FtAQP13*. Methyl jasmonate (MeJA) responsive elements were predominantly found in genes like *FtAQP12* and *FtAQP6*. The promoter regions also contained elements responsive to auxin, gibberellin, and salicylic acid.

Additionally, the *FtAQP* promoters are enriched with multiple stress-related elements, which may be associated with plant responses to environmental adversities. Low-temperature responsive (LTR) elements were mainly distributed in *FtAQP20*, *FtAQP21*, and *FtAQP22*. Defense and stress-responsive elements (TC-rich repeats) were observed in *FtAQP20*, *FtAQP25*, *FtAQP14*, and *FtAQP16*. Most gene promoters contained anaerobic induction elements (AREs), which were prominently distributed in *FtAQP13*. Elements potentially involved in circadian control were also identified in members such as *FtAQP17*, *FtAQP23*, *FtAQP25*, and *FtAQP7*.

### 3.6. Synteny Analysis of the FtAQP Gene Family

**Figure 5:**
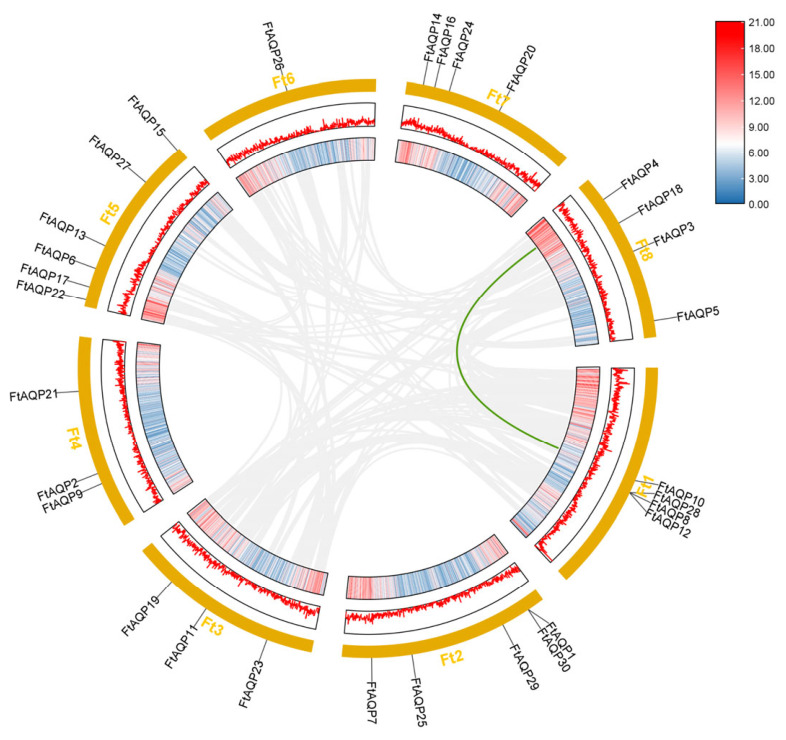
Synteny analysis of the *FtAQP* gene family. Collinearity analysis was performed using the TBtools software.

To investigate gene duplication events, an intra-species collinearity analysis of the *FtAQP* genes within the Tartary buckwheat genome was performed using TBtools (Figure 5). The results revealed only one collinear gene pair: *FtAQP4* located on Chromosome 8 and *FtAQP10* located on Chromosome 1. Combined with the phylogenetic analysis, this syntenic relationship indicates that this gene pair is likely the result of a chromosomal segmental duplication event. Furthermore, it is speculated that these genes existed prior to the relevant divergence events and have successfully maintained their collinear relationship throughout evolution.

**Figure 6:**
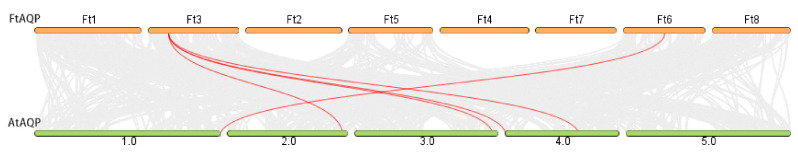
An interspecific collinearity analysis of the *AQP* gene family members between Tartary buckwheat and *A. thaliana* revealed five collinear gene pairs.

Figure 6 displays the syntenic relationships between *F*. *tataricum* and *A*. *thaliana*. The links between *FtAQP* genes and their *Arabidopsis* orthologs suggest a high degree of evolutionary conservation in genomic organization between these two species.

### 3.7. Tissue-Specific Expression Patterns of the FtAQP Genes

**Figure 7:**
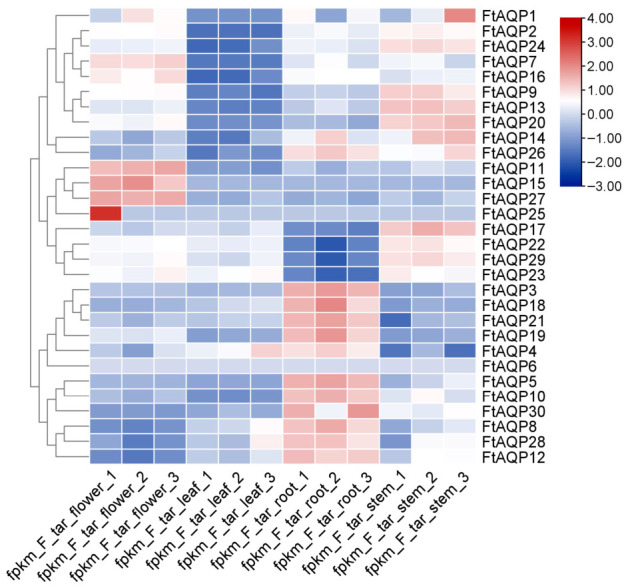
Tissue-specific expression patterns of the *FtAQP* gene family. The heatmap illustrates the expression profiles of *FtAQP* genes across four different tissues of Tartary buckwheat: flowers, leaves, roots, and stems (each with three biological replicates). The color scale on the right represents the relative expression levels, with red indicating high transcription levels and blue indicating low transcription levels. A hierarchical clustering dendrogram of the genes is displayed on the left.

To investigate the expression patterns of the *FtAQP* gene family across different tissues (flowers, leaves, roots, and stems), RNA-seq data were retrieved from the NCBI database and visualized as a heatmap using TBtools (Figure 7). The heatmap revealed significant spatial expression heterogeneity among the *FtAQP* members across different organs.

Notably, *FtAQP27* exhibited highly specific enrichment in flowers. A large subset of genes, including *FtAQP3*, *18*, *21*, *19*, *5*, *10*, *30*, *8*, *28*, and *12*, showed significantly high expression levels in root tissues, whereas their transcription levels were relatively low in leaves and flowers. Conversely, genes such as *FtAQP9*, *13*, and *20* displayed robust expression in stems.

### 3.8. qRT-PCR Analysis of the FtAQP Genes

**Figure 8:**
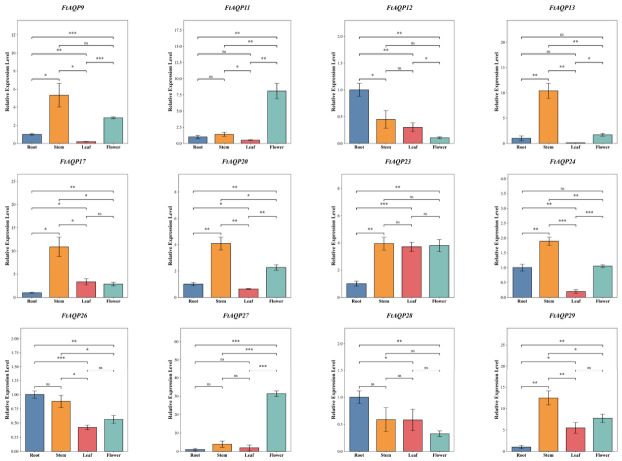
qRT-PCR analysis of *FtAQP* genes in various organs. Relative expression of 12 selected *FtAQP* members across roots, stems, leaves, and flowers. Error bars indicate the standard deviation of three biological replicates. Statistical significance among different tissues was determined using one-way analysis of variance (ANOVA) followed by Tukey’s honestly significant difference (HSD) post hoc test. Significance levels were defined as follows: * *p* < 0.05, ** *p* < 0.01, *** *p* < 0.001, and ‘ns’ indicates not significant.

To further investigate the tissue-specific expression profiles and potential functional divergence of the *FtAQP* genes across different organs, 12 representative members (*FtAQP9*, *11*, *12*, *13*, *17*, *20*, *23*, *24*, *26*, *27*, *28*, and *29*) were selected from the 30 *FtAQP* genes to evaluate their tissue-specific expression patterns using qRT-PCR (Figure 8). As the stem acts as the central hub for long-distance water transport, *FtAQP9*, *13*, *17*, *20*, and *24* all exhibited significantly elevated expression levels in stem tissues. Notably, the relative expression levels of *FtAQP13* and *FtAQP17* were exceptionally high, demonstrating highly significant differences when compared to other organs.

Furthermore, the transcription level of *FtAQP11* was significantly up-regulated in floral organs compared to roots. In stark contrast, *FtAQP11* displayed markedly low expression in leaf tissues, reflecting a significant divergence in the expression regulatory mechanisms of this gene between reproductive and vegetative organs. Although many genes tended to be preferentially expressed in the aerial parts (shoots), *FtAQP12* and *FtAQP28* consistently maintained robust and stable transcription levels in the roots, significantly surpassing their expression in leaves and flowers.

To validate the reliability of the RNA-seq transcriptomic data, a Pearson correlation analysis was performed between the qRT-PCR relative expression levels of the 12 representative *FtAQP* genes across various tissues and their corresponding RNA-seq profiles (Supplementary File Figure S1). The analysis revealed a highly significant positive correlation between the expression levels detected by the two techniques (Pearson’s r = 0.846, *p* < 0.01). The spatiotemporal expression trends demonstrated by both methods were in complete agreement, thereby confirming the reliability of our transcriptomic data and providing observational support for our hypothesized spatial water management models.

## 4. Discussion

### 4.1. Expansion and Structural Conservation of the FtAQP Gene Family

Whole-genome duplication (WGD) events experienced during species evolution are often the primary drivers of gene family expansion. In this study, we identified only 30 *FtAQP* genes in Tartary buckwheat. Compared to crops that have undergone complex polyploidization events, such as wheat (113) or oat (45), the AQP family size in Tartary buckwheat appears quite streamlined, more closely resembling that of the diploid model plant *A. thaliana* (35) [14,15,30]. This disparity in gene family size suggests that Tartary buckwheat may not have adopted a “quantity-driven” evolutionary route to cope with extreme environments such as drought.

Instead, the 12 pairs of paralogous genes (identified via phylogenetic analysis), along with the highly conserved transmembrane helices and NPA motifs, imply that Tartary buckwheat exhibited a tendency to retain core genetic resources during its evolution. In harsh, cold, and arid environments, maintaining a massive genome would undoubtedly consume excessive metabolic energy. Therefore, Tartary buckwheat appears to have adopted a more economical adaptive strategy: relying on the subfunctionalization of a few core genes to finely regulate water balance, rather than simply accumulating gene copies. This conservative evolutionary approach provides a crucial theoretical basis for how Tartary buckwheat maintains water homeostasis under complex environmental conditions.

### 4.2. Multi-Dimensional Regulation of Water Transport by Cis-Acting Elements

Gene expression patterns are largely determined by cis-acting elements in their promoter regions. We identified numerous elements related to light responsiveness, phytohormones, and abiotic stresses in the promoters of *FtAQP* genes, providing clues to how Tartary buckwheat perceives and responds to complex environments. For instance, the widespread distribution of light-responsive elements across all members not only reflects the close connection between aquaporins and processes like photosynthesis and stomatal movement, but also aligns well with the ecological characteristics of Tartary buckwheat’s native habitat (e.g., high altitude and intense solar radiation).

Furthermore, abscisic acid (ABA) serves as a core phytohormone in plant drought responses, and its responsive elements are particularly dense in *FtAQP* genes highly expressed in roots and stems (e.g., *FtAQP10*, *FtAQP30*). This suggests that under soil water deficit, these specific aquaporins might be directly regulated by ABA signaling pathways, subsequently participating in systemic osmotic adjustment. Meanwhile, the presence of elements such as LTR (low-temperature responsiveness) and ARE (anaerobic induction) further implies that the *FtAQP* family functions not merely as simple water channels. Rather, they may act as vital molecular hubs that integrate plant responses to multiple environmental stresses, including chilling and waterlogging.

### 4.3. Tissue-Specific Expression Heterogeneity and Water Coordination Strategies

Efficient water utilization in plants relies on coordinated management at the whole-plant level, which is well exemplified by the expression heterogeneity of the *FtAQP* family across different tissues. As the “source” for water uptake, the synchronized high expression of multiple *FtAQP* genes in the roots likely facilitates the maintenance of basal transmembrane water uptake driving forces, even under fluctuating soil water potentials. As water enters the stem—the long-distance transport system—genes such as *FtAQP13*, *FtAQP17*, and *FtAQP20* are significantly upregulated. This transcriptionally active state may help enhance the lateral flow of water in the xylem, thereby maintaining the osmotic pressure of the vascular system and mitigating the risk of vessel embolism.

During the reproductive development stage, genes like *FtAQP27* exhibit extremely high tissue specificity in floral organs. Given that reproductive organs are highly sensitive to water fluctuations, this specific enrichment suggests a specialized role in local osmotic regulation, such as during pollen development or stigma hydration, to potentially facilitate successful pollination.

Notably, in contrast to the active transcription observed in roots and stems, most *FtAQPs* exhibit a distinct downward trend in expression within leaves. From a plant physiological perspective, this likely represents an active defense strategy of Tartary buckwheat against drought. By reducing the abundance of aquaporins on the plasma membranes of mesophyll and guard cells, the plant decreases cell membrane water permeability. This passive water retention mechanism helps maintain a relatively high intracellular water potential under intense transpiration pull. This spatially differentiated expression pattern—optimizing water acquisition and transport in roots and stems while restricting water loss in leaves—constitutes a compelling predictive model for the molecular basis underlying the exceptional drought tolerance of Tartary buckwheat.

## 5. Conclusions

In this study, we identified 30 *FtAQP* genes in the Tartary buckwheat genome. Compared to polyploid crops like wheat, the AQP family in Tartary buckwheat is relatively small. This suggests that rather than simply accumulating gene copies, Tartary buckwheat evolved a “metabolic economics” strategy to cope with drought. It achieves this by retaining core paralogous genes and driving their functional divergence at the transcriptional level.

Regarding whole-plant water management, we observed a clear division of labor among these genes across different organs. Specifically, the robust expression of multiple genes in the roots ensures efficient initial water uptake. As water moves into the stem, the activation of specific *FtAQPs* facilitates long-distance transport and prevents vessel embolism. During reproduction, exclusively enriched members in floral organs manage local osmotic homeostasis. Interestingly, most *FtAQPs* are transcriptionally suppressed in leaves, reflecting an active “isohydric behavior” to minimize water loss under stress.

Furthermore, promoter analyses indicate that these aquaporins are not merely physical pores; they act as vital molecular hubs that integrate photoperiod, ABA, and various abiotic stress signals. Overall, this study clearly uncovers the molecular mechanisms underlying the exceptional drought tolerance of Tartary buckwheat. These findings also provide specific genetic targets for future efforts to breed climate-resilient crops with improved water use efficiency (WUE) through the precise manipulation of aquaporins.

## Figures and Tables

**Table 1:** Real-time quantitative PCR primers.

Name	Forward Primer (5′-3′)	Reverse Primer (5′-3′)
*FtActin*	TGCTTTGAGAACTGGAGCGG	TTCGAGCTCTTCGAGTTCCG
*FtAQP9*	AGGACTAGGCTGCCCACTTA	GCGAAGCACGTATGTGTGAC
*FtAQP11*	TGGCTTGACTACTCCAGTGC	AACGTTGGCCCCAACTACAA
*FtAQP12*	ATTCGTGGCAAGGAAGGTGT	AGTGCCGATTATCTCCGCTG
*FtAQP13*	CAGGTGGACACGTCAATCCA	TCACTCCACTTGCTACGCTG
*FtAQP17*	TCAACCCAGCTGTCACCTTC	AATGCGTTGGTTGCTGACAC
*FtAQP20*	TGGAGCAAGCATTTAGCCGA	GTCGGACTCGGAAGTAGTGC
*FtAQP23*	TGCTTGGGCTTTTGGTGGTA	GACCACACCTGCACCACATA
*FtAQP24*	AATCACACTTGCTAGATGCCA	AGATCGGTTGCTGTCTGGAA
*FtAQP26*	GAGAGCTGGCAGGTATAGCG	GTACACTCCGGCACCAGAAA
*FtAQP27*	GACGGCTTTGATCGCAACTC	TCGCCTCCATTATCGCCATC
*FtAQP28*	GCGTAACGCTAGAGACTCCC	CACCATTGGACCAACCCAGA
*FtAQP29*	TATCCGGCCACCCAATTCAC	TGGCTGTATGGTGCTAACGA

**Table 2:** Physicochemical properties of the FtAQP protein family.

Gene Name	Gene ID	Amino Acids Length/aa	Molecular Weight/Da	PI	Total Number of Negatively Charged Residues	Total Number of Positively Charged Residues	Instability Index	Aliphatic Index	Grand Average of Hydropathicity
*FtAQP1*	FtPinG0005551300.01	260	26,992.57	7.86	13	14	29.12	106.62	0.622
*FtAQP2*	FtPinG0009190200.01	278	29,419.23	9.18	14	20	26.68	101.08	0.492
*FtAQP3*	FtPinG0002644000.01	280	29,713.45	7.72	18	19	28.42	104.29	0.551
*FtAQP4*	FtPinG0009223600.01	250	25,584.65	4.93	14	8	25.81	113.64	0.837
*FtAQP5*	FtPinG0007225000.01	286	30,493.4	7.67	19	20	27.66	106.78	0.518
*FtAQP6*	FtPinG0001353400.01	252	26,362.71	5.14	14	8	28.46	118.89	0.875
*FtAQP7*	FtPinG0003646600.01	251	25,777.8	5.9	11	7	24.82	106.61	0.788
*FtAQP8*	FtPinG0006769900.01	198	20,842.33	9.77	5	14	26.74	107.93	0.614
*FtAQP9*	FtPinG0004904200.01	285	30,416.37	8.88	15	19	30.77	99.33	0.454
*FtAQP10*	FtPinG0008427100.01	249	25,577.66	5.33	12	7	22.97	110.96	0.874
*FtAQP11*	FtPinG0004531000.01	249	26,003.54	5.78	14	10	22.41	119.08	0.833
*FtAQP12*	FtPinG0009899700.01	282	30,099.87	8.34	18	20	28.11	104.86	0.47
*FtAQP13*	FtPinG0008073200.01	249	25,361.4	5.9	12	8	33.46	109.72	0.797
*FtAQP14*	FtPinG0002889000.01	287	30,739.82	8.87	17	21	32.6	99.34	0.418
*FtAQP15*	FtPinG0004697900.01	253	26,104.07	4.94	14	7	25.93	111.9	0.793
*FtAQP16*	FtPinG0009506300.01	244	26,160.23	9.84	8	19	24.45	103.16	0.728
*FtAQP17*	FtPinG0005720300.01	252	25,855.95	5.7	12	7	21.36	111.63	0.823
*FtAQP18*	FtPinG0008253300.01	311	32,415.56	8.81	17	20	35.82	96.66	0.407
*FtAQP19*	FtPinG0009619600.01	305	31,581.74	8.22	17	19	40.92	98.82	0.507
*FtAQP20*	FtPinG0007255200.01	282	29,763.55	9.06	14	19	39.48	99.04	0.545
*FtAQP21*	FtPinG0006228900.01	287	30,675.74	8.84	17	21	35.36	100.35	0.454
*FtAQP22*	FtPinG0000553000.01	251	26,028.13	5.18	15	8	29.71	108.92	0.757
*FtAQP23*	FtPinG0001689700.01	285	30,577.53	9.17	14	20	31.05	96.25	0.365
*FtAQP24*	FtPinG0004150100.01	286	30,581.55	8.87	17	21	36.18	100.35	0.421
*FtAQP25*	FtPinG0005321900.01	256	27,063.44	6.58	15	14	35.28	108.67	0.534
*FtAQP26*	FtPinG0006007500.01	307	31,699	7.73	17	18	33.58	99.54	0.505
*FtAQP27*	FtPinG0003266800.01	243	25,660.55	8.94	12	16	24.12	110.45	0.827
*FtAQP28*	FtPinG0006770100.01	286	30,370.21	8.89	16	20	28.89	105.77	0.481
*FtAQP29*	FtPinG0000824700.01	283	29,999.69	9.05	15	20	39.16	97.28	0.449
*FtAQP30*	FtPinG0005551100.01	288	29,894.95	8.87	14	17	32.72	103.09	0.514

**Table 3:** Predicted secondary structures of the *FtAQP* gene family members.

Gene	Gene ID	α-Helix (%)	Extension Chain (%)	β-Turn (%)	Random Coil
*FtAQP1*	FtPinG0005551300.01	30.77	25	9.62	34.62
*FtAQP2*	FtPinG0009190200.01	31.29	26.98	9.71	32.01
*FtAQP3*	FtPinG0002644000.01	37.86	21.79	10.36	30
*FtAQP4*	FtPinG0009223600.01	30	31.6	10	28.4
*FtAQP5*	FtPinG0007225000.01	39.16	21.68	8.39	30.77
*FtAQP6*	FtPinG0001353400.01	33.73	28.97	10.71	26.59
*FtAQP7*	FtPinG0003646600.01	23.51	35.46	9.96	31.08
*FtAQP8*	FtPinG0006769900.01	31.82	28.28	8.59	31.31
*FtAQP9*	FtPinG0004904200.01	28.07	22.11	9.12	40.7
*FtAQP10*	FtPinG0008427100.01	31.73	31.73	10.44	26.1
*FtAQP11*	FtPinG0004531000.01	30.92	28.11	9.64	31.33
*FtAQP12*	FtPinG0009899700.01	35.11	24.47	8.87	31.56
*FtAQP13*	FtPinG0008073200.01	25.7	28.11	12.85	33.33
*FtAQP14*	FtPinG0002889000.01	33.1	19.51	10.8	36.59
*FtAQP15*	FtPinG0004697900.01	32.02	32.02	8.3	27.67
*FtAQP16*	FtPinG0009506300.01	39.34	26.23	11.07	23.36
*FtAQP17*	FtPinG0005720300.01	29.76	28.97	10.32	30.95
*FtAQP18*	FtPinG0008253300.01	29.26	20.58	8.04	42.12
*FtAQP19*	FtPinG0009619600.01	33.77	18.36	6.89	40.98
*FtAQP20*	FtPinG0007255200.01	35.82	21.63	9.57	32.98
*FtAQP21*	FtPinG0006228900.01	28.92	22.65	9.76	38.68
*FtAQP22*	FtPinG0000553000.01	31.47	28.29	10.36	29.88
*FtAQP23*	FtPinG0001689700.01	23.16	26.32	9.47	41.05
*FtAQP24*	FtPinG0004150100.01	27.62	22.03	10.49	39.86
*FtAQP25*	FtPinG0005321900.01	37.11	24.22	9.77	28.91
*FtAQP26*	FtPinG0006007500.01	27.04	23.13	8.79	41.04
*FtAQP27*	FtPinG0003266800.01	50.62	20.58	7.41	21.4
*FtAQP28*	FtPinG0006770100.01	33.92	25.52	8.74	31.82
*FtAQP29*	FtPinG0000824700.01	27.92	25.44	9.19	37.46
*FtAQP30*	FtPinG0005551100.01	33.33	22.57	10.76	33.33

## Data Availability

The data presented in this study are available on request from the corresponding author. The data are not publicly available due to ethical reason.

## Supplementary Materials

The following supporting information can be downloaded at https://www.mdpi.com/article/10.3390/genes17040479/s1: Figure S1: Correlation between RNA-seq and gRT-PCR.
