# Supplementary figures and images for: Genome-Wide Identification and Tissue-Specific Expression Analysis of the FtAQP Gene Family in Tartary Buckwheat (Fagopyrum tataricum)
Source: Genes (Basel). 2026 Apr 17;17(4):479. doi: 10.3390/genes17040479 (PMC13116170; doi:10.3390/genes17040479)

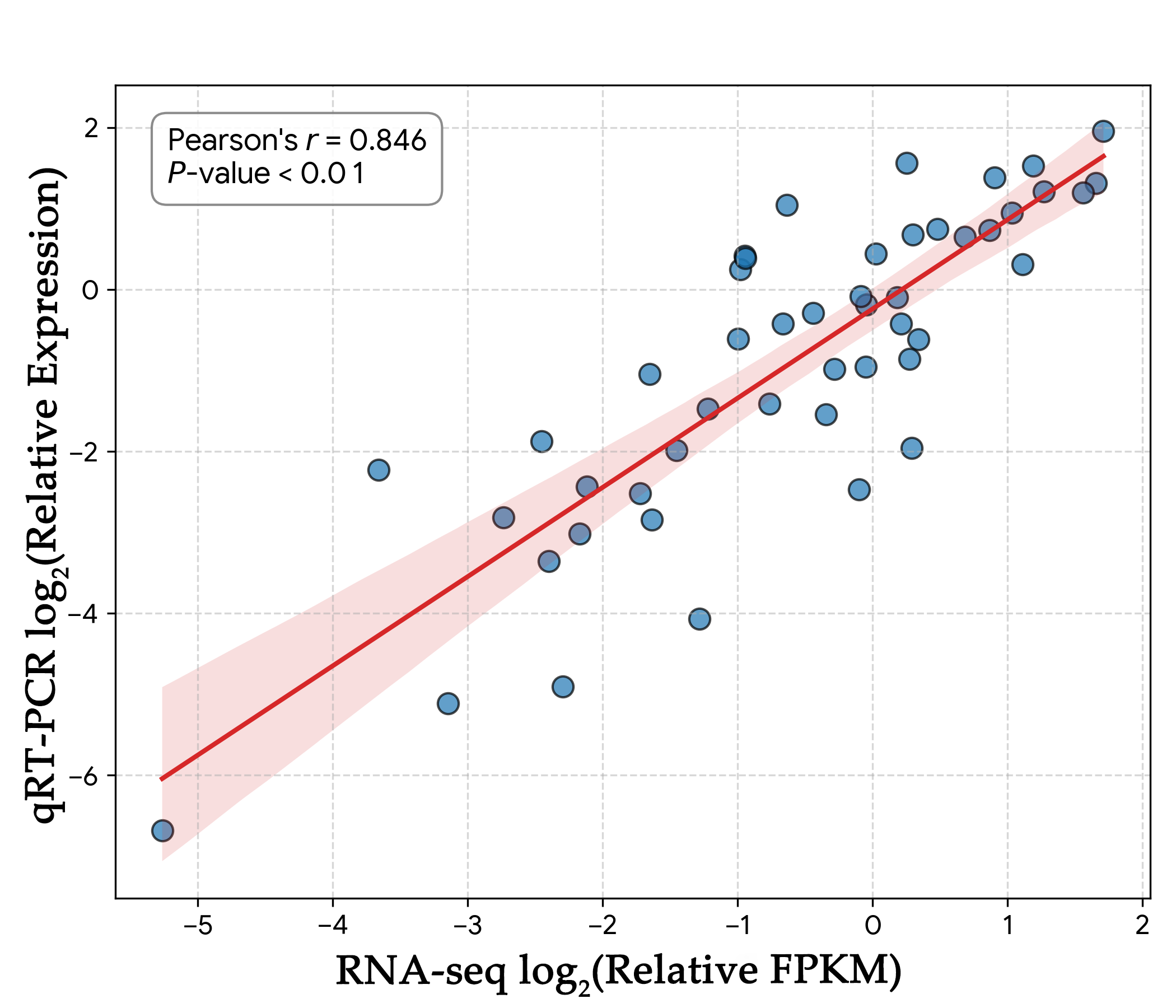

Supplement: Supplementary file 1 [file genes-17-00479-s001.zip › genes-4244917-supplementary.png]
